# Prediction of Productivity Costs Related to Cervical Cancer Mortality in Indonesia 2018

**DOI:** 10.21315/mjms2022.29.1.13

**Published:** 2022-02-23

**Authors:** Susi Ari Kristina, Dwi Endarti, Hardika Aditama

**Affiliations:** Department of Pharmaceutics, Faculty of Pharmacy, Universitas Gadjah Mada, Yogyakarta, Indonesia

**Keywords:** cervical cancer, productivity cost, years of life lost, Indonesia

## Abstract

**Background:**

Cervical cancer is the second leading cause of death in Indonesia, causing a significant societal burden. This study aims to quantify the burden of cervical cancer in terms of years of life lost (YLL) and productivity cost to support the idea that cervical cancer has substantial economic implications.

**Methods:**

Using an epidemiological approach on the prevalence data of 2018, the productivity cost and YLL were estimated by calculating the number of cervical cancer deaths, life expectancy, annual earnings and employment participation rate. Cervical cancer mortality data were obtained from the Global Cancer Observatory (GLOBOCAN) 2018, life expectancy for Indonesia from the WHO Life Tables (2019), and the annual earnings and participation rate of Indonesia were retrieved from the National Statistics Bureau (2018).

**Results:**

In 2018, there were 17,253 deaths due to cervical cancer in Indonesia, resulting in 246,350 YLL with a total productivity cost of Indonesian Rupiah (IDR)23,174 trillion. The age group of 50 years old–64 years old experienced the greatest loss of earnings (IDR12,149 trillion), followed by the 35 years old–49 years old (IDR8,944 trillion) and 20 years old–34 years old (IDR8,944 trillion) age groups.

**Conclusion:**

The productivity impact of loss of earning due to cervical cancer mortality is significant. This information may assist decision makers in allocating scarce resources among competing priorities.

## Introduction

Cancer is one of the leading causes of death worldwide. In 2018, the World Health Organization (WHO) reported cancer as the second most common cause of death, responsible for approximately 9.6 million deaths (about one in six) worldwide (1), of which 70% occur in low- and-middle income countries (LMICs) (2). In 2012, the economic burden due to cancer in the LMICs was 0.33% of their combined GDP (3); even developed countries like China reported a total loss of USD28 billion due to cancer (4). As reported by WHO in 2018, lung cancer was the leading cause of most total productivity losses in Brazil, Russia and South Africa, followed by liver cancer in China and lip and oral cavity cancer in India (4). Likewise, the International Agency for Research on Cancer (IARC), in their 2018 report, described 348,809 cases of cancer and 207,210 cancer deaths in Indonesia, of which lung cancer, breast cancer and cervical cancer were the foremost reasons for cancer-related mortality (1).

Every year, more than 2 million women are diagnosed with breast or cervical cancer around the world (5, 6). Cervical cancer is the third most common cancer in women globally, with an estimated 569,847 new cases and 311,365 deaths worldwide as reported in 2018. In Indonesia, cervical cancer ranks as the second most frequent cancer endangering the country’s 93.15 million female population; about 18,279 deaths occur annually due to cervical cancer in Indonesia (7, 8). Several factors contribute to the etiology of cervical cancer, of which 87% can be attributed to the human papillomavirus (HPV) (9).

Multiple studies have described the significant economic impact of cancer in LMICs compared to high-income nations — a population-based study in 2018 about the economic impact of cervical cancer in sub-Saharan Africa reported an estimated total annual cost of cervical cancer as USD19 million (10). Likewise, the total economic cost of colorectal cancer in Vietnam was Vietnamese Dong (VND)3,041.88 billion (USD132.9 million), with indirect costs accounting for 83.58% of the total cost and 82.61% of which is future income loss (11). Notably, a Malaysian study investigating the prevalence of cancers and their corresponding risk factors reported that lung cancer, laryngeal cancer, and esophageal cancer were the three most predominant cancers and tobacco smoking was the most prevalent contributing risk factor across genders (12). Furthermore, in Thailand, productivity loss due to premature deaths and absenteeism caused by smoking-related diseases was a massive Thai Baht (THB)62.24 billion (USD1.82 billion). Overall, the cost of smoking accounted for 18.19% of the total health expenditure in 2016 (13). Therefore, understanding the economic and health burden of the disease is essential for decision-making and budget planning by the public health authorities.

Owing to the substantial prevalence of cervical cancer, the consequent associated economic burden is significant both on society and the individual. However, there is a dearth of studies that have analysed the cost of cervical cancer, especially in Indonesia, where cervical cancer-related mortality is significant. A previous study in 2018 estimated the economic burden of cancer related to smoking in Indonesia at 221,384 years lived with disability (YLD) (14). Another study focusing on cancer due to passive smoking in Indonesia showed that the indirect cost associated with premature death (ICPD) was Indonesian Rupiah (IDR)1,832 trillion (15).

Every person who is unable to work due to cancer, either temporarily or permanently, constitutes an economic loss to society. The most notable measures to quantify the burden of cancer are years of life lost (YLL) and productivity cost; productivity costs provide an estimation of the country’s loss due to cancer-related mortality. These measures provide strong evidence to the concerned authorities about the associated economic impact (16); however, such data for Indonesia, especially the productivity costs, is scarce. The available studies evaluating the economic impact of cervical cancer in Indonesia have only described it from the perspective of the healthcare providers (14–15) and concluded that cervical cancer has a substantial economic impact (17). A country’s health policies are focused on controlling and reducing the total economic burden of cancer; therefore, the current study aims to examine the estimated productivity costs related to cervical cancer mortality in Indonesia. Since ICPD account for a considerable fraction of the total economic burden of HPV-related diseases, they are also included in our research.

## Methods

To estimate the overall burden of cervical cancer mortality in Indonesia, the study involved three broad steps: i) collecting data for cervical cancer mortality; ii) computing the YLL as the product of the number of cervical cancer deaths and life expectancy; and iii) estimating the productivity costs related to cervical cancer mortality by multiplying the number of cervical cancer deaths by employment participation rate and annual earnings.

### Data Sources

Using the standard methodology for measuring the burden of a disease, the statistics for cancer mortality in Indonesia for the year 2018 were retrieved from the Global Cancer Observatory (GLOBOCAN) 2018 report (1) and life expectancy data for an average Indonesian female were taken from the WHO Life Tables for 2019 (18). Lastly, the annual earnings and participation rate data for the Indonesian population were retrieved from the National Statistics Bureau’s 2018 report.

### Years of Life Lost for Cervical Cancer Mortality in Indonesia in 2018

The YLL value was calculated by multiplying the number of cervical cancer deaths with number of years left to live as calculated from the overall life expectancy, using the formula:


YLL=N×L

Where ‘*N*’ denotes the number of cervical cancer deaths (in different age groups: 20 years old–34 years old, 35 years old–49 years old, 50 years old–64 years old and 65 years old–79 years old) and ‘L’ denotes the number of years remaining to live as calculated from the life expectancy (19).

### Mean of Years Life Lost for Cervical Cancer Mortality in Indonesia in 2018

The mean of YLL for cervical cancer mortality was obtained by dividing YLL by the number of deaths due to cervical cancer. This parameter provides a measure of the burden of cervical cancer for the individual patient rather than the population as a whole. The mean of YLL shows how much a patient’s life is likely to be shortened by their cervical cancer (20).

### Productivity Costs Related to Cervical Cancer Mortality in Indonesia in 2018

This value was obtained by multiplying the number of cervical cancer deaths (in different age groups: 20 years old–34 years old, 35 years old–49 years old, 50 years old–64 years old and 65 years old–79 years old) with the employment participation rate and annual earnings (16). The annual income and participation rate for an Indonesian female were retrieved from National Statistics Bureau 2018 (21). The total loss of earnings due to cervical cancer mortality in Indonesia as calculated for the year 2018 was also converted to USD using the conversion rate for IDR to USD provided by the Bank of Indonesia (12).

All data processing and calculations were done using Microsoft Excel.

## Results

Based on the collected data, it was observed that in the year 2018, there were 17,253 deaths due to cervical cancer in Indonesia; the YLL values for cervical cancer are presented in Table 1. As evident from Table 1, cervical cancer mortality in Indonesia in 2018 accounted for a total of 246,350 YLL. The highest number of YLL were seen in the 50 years old–64 years old of age group (112,952 YLL), followed by 35 years old–49 years old (112,520 YLL) and 20 years old–34 years old (13,376 YLL). These numbers of Indonesian YLL are indicative of the substantial loss of potential productivity due to cervical cancer mortality. The highest mean of YLL of cervical cancer was seen in the 20 years old–34 years old of age group (44 mean of YLL), followed by the 35 years old–49 years old, 50 years old–64 years old and the 65 years old–79 years old of age groups.

Table 2 presents the data for productivity costs related to cervical cancer mortality in Indonesia in 2018. The total loss of earning because of deaths by cervical cancer in 2018 was a massive IDR23,174 trillion. The highest loss of earning was seen in the age group of 50 years old–64 years old (IDR12,149 trillion) which also had the highest cervical cancer mortality, followed by the 35 years old–49 years old (IDR8,944 trillion) and 20 years old–34 years old (IDR12,149 trillion) of age groups.

## Discussion

This is the first study to evaluate the societal costs and burden of premature mortality caused by cervical cancer in Indonesia using the representative measures of mortality rate, potential YLL and productivity cost. There were 17,253 cervical cancer deaths in Indonesia in 2018, accounting for 246,350 YLL in total. The total loss of earnings due to cervical cancer mortality during this period was IDR23,174 trillion (USD1,716 million). Our findings are in agreement with previous studies that reported that the cost of cervical cancer mortality varied by region and country. Ngcamphalala et al. (10) reported considerable costs of cervical cancer in Eswatini, with indirect costs accounting for 27% (USD5.3 million) of total expenditures and productivity loss due to premature mortality accounting for 67% (USD3.5 million). Likewise, a Vietnamese study describing the burden of colorectal cancer reported that the productivity costs of the cancer were about 83.58% of the overall cost, with 82.61% of future income loss occurring during the productive years (11).

The estimation of productivity cost is greatly influenced by working-age individuals and their average earnings (16). Despite the availability of many prophylactic methods, HPV-related cancers continue to be a significant cause of death in many regions of the world, especially LMICs. Cervical cancer mortality and morbidity are high in countries where the HPV vaccine is not yet obligatory, such as Indonesia (6). And, studies from these developing countries report that cancer-related productivity loss causes about 60%–85% of the economic burden (4, 11, 14, 15).

We could not find any studies that calculated the productivity costs of cervical cancer mortality in Indonesia. However, one study had described specific factors, like smoking, that affect the productivity costs due to cervical cancer mortality (8). They reported that about 77% of YLL among females was due to oral, lung, and cervical cancers and mortality caused by cervical cancer due to smoking accounted for 5,360 YLL, with the total loss of earning of USD2,556,821 (8). Besides smoking, the biggest burden of disease associated with passive smoke in Indonesia was due to lung cancer, accounting for IDR1,075 trillion, which is over half of the total disease burden value (IDR2,665 trillion) (22). In Australia, the productivity costs of premature mortality were estimated by deriving the Present Value of Lifetime Income (PVLI), which represents the lifetime stream of private income an individual is expected to earn valued in the present-day currency. The PVLI lost due to cervical cancer in Australia in 2013 was estimated at USD39 million (23). In central-eastern Europe, cervical cancer was among the five leading cancer sites with high premature mortality, costing about €400 million (17). In 2000, it was estimated that there were 37,594 women dying from cervical cancer in the US who would have otherwise received earnings through labour force participation, representing 28.8% of the total cohort of women dying from cervical cancer who would have otherwise been alive during that year (16). Consequently, the total productivity loss due to cervical cancer mortality in that year was estimated at USD1.3 billion. A vast majority of this productivity loss (72%) was anticipated to occur among the women aged between 40 years old and 64 years old in the year 2000 (16).

When we compared the burden of cervical cancer as calculated in our study to that of other cancers, such as lung cancer, we discovered that cervical cancer had surpassed other cancers by a significant proportion. The total number of disability-adjusted life-years (DALYs) lost to lung cancer in Australia, the Philippines and Singapore were estimated as 91,695; 38,584 and 12,435, respectively. In these countries, the YLL or premature death due to cervical cancer accounted for more than 95% of DALYs (24). The high number of YLL and productivity losses caused by cervical cancer in Indonesia necessitate an increase in cervical cancer treatment costs at the national level and governments must be made aware of these issues to avoid adding to the community’s burden. Based on our results, we suggest that ample efforts be directed toward prevention and promotion strategies for cervical cancer, such as HPV vaccination, pap smears or HPV testing, as well as public health awareness campaigns.

This is the first national study to estimate YLL and productivity cost of cervical cancer in Indonesia and provide a comprehensive insight to policymakers of the burden of cervical cancer. However, there were some limitations in the study. It is noteworthy that besides mortality, cancer is also responsible for significant morbidity with associated productivity costs, including the need for time away from work for both the patient and their caregivers. Our analysis estimated the productivity-related costs of cancer mortality alone. Therefore, future studies should include aspects of cancer morbidity as well to fully gauge the productivity burden of cancer.

## Conclusion

The productivity impact of loss of earning due to cervical cancer mortality is significant, resulting in a loss of 246,350 YLL and IDR23,174 trillion (USD1,716 million) loss of earning in Indonesia in a single year (2018). The magnitude of loss demonstrates the extent of the societal burden of cancer and the potential economic gains that could be achieved through investment in effective interventions. This information may assist decision makers in allocating scarce resources among competing priorities. Cervical cancer typically presents late in Indonesia, with a high associated mortality rate, and HPV screening and vaccination can potentially reduce cervical cancer mortality. Governments should adopt these strategies as a central component of health policy along with addressing the general population’s educational and training needs.

## Figures and Tables

**Table 1:** Mortality data and YLL for cervical cancer in Indonesia in 2018

Age groups (years old)	Number of deaths	YLL*	Mean of YLL
20–34	304	13,376	44
35–49	3,880	112,520	29
50–64	8,068	112,952	14
65–79	5,001	7,502	2
Total	17,253	246,350	–

Note:

* YLL was calculated based on the WHO Life Table 2019

**Table 2:** Total loss of earnings due to cervical cancer mortality in Indonesia in 2018

Age groups (years old)	Employment participation rate (%)*	Annual earnings (in thousand IDR)	Total loss of earnings due to mortality (in thousand IDR)	Total loss of earning due to mortality (in USD)**
20–34	73.23	32,400	721,286,208	53,428,608
35–49	71.15	32,400	8,944,408,800	662,548,800
50–64	54.56	27,600	12,149,246,208	899,944,164
65–79	15.10	18,000	1,359,271,800	100,686,800

Total	–	–	23,174,213,016	1,716,608,372

Notes:

* Based on National Statistics Bureau Data;

** conversion rate IDR to USD as provided by the Bank of Indonesia
